# Versatile magnetic configuration for the control and manipulation of superparamagnetic nanoparticles

**DOI:** 10.1038/s41598-023-32299-9

**Published:** 2023-03-31

**Authors:** Alessandro Surpi, Tatiana Shelyakova, Mauro Murgia, José Rivas, Yolanda Piñeiro, Pierpaolo Greco, Milena Fini, Valentin Alek Dediu

**Affiliations:** 1 Istituto per lo Studio dei Materiali Nanostrutturati (CNR-ISMN), 40129 Bologna, Italy; 2 IRCCS Istituto Ortopedico Rizzoli, SC Scienze e Tecnologie Chirurgiche, 40136 Bologna, Italy; 3 Italian Institute of Technology, Center for Translational Neurophysiology (IIT), 44121 Ferrara, Italy; 4 Laboratorio de Nanomagnetismo y Nanotecnologia, Departamento de Fisica Aplicada, Istituto NANOMAG, Universitade de Santiago de Compostela, 15782 Santiago de Compostela, Spain; 5 Dipartimento di Neuroscienze e Riabilitazione, Università di Ferrara, 44121 Ferrara, Italy

**Keywords:** Materials science, Nanoscience and technology, Physics

## Abstract

The control and manipulation of superparamagnetic nanoparticles (SP-MNP) is a significant challenge and has become increasingly important in various fields, especially in biomedical research. Yet, most of applications rely on relatively large nanoparticles, 50 nm or higher, mainly due to the fact that the magnetic control of smaller MNPs is often hampered by the thermally induced Brownian motion. Here we present a magnetic device able to manipulate remotely in microfluidic environment SP-MNPs smaller than 10 nm. The device is based on a specifically tailored configuration of movable permanent magnets. The experiments performed in 500 µm capillary have shown the ability to concentrate the SP-MNPs into regions characterized by different shapes and sizes ranging from 100 to 200 µm. The results are explained by straightforward calculations and comparison between magnetic and thermal energies. We provide then a comprehensive description of the magnetic field intensity and its spatial distribution for the confinement and motion of magnetic nanoparticles for a wide range of sizes. We believe this description could be used to establish accurate and quantitative magnetic protocols not only for biomedical applications, but also for environment, food, security, and other areas.

## Introduction

Magnetic nanoparticles (MNP) have found multiple uses across a wide spectrum of biomedical applications^1,2^ such as hyperthermia for cancer treatment^3^, remotely controlled drug delivery^4^ and others both in-vivo^5,6^ and in-vitro^7–9^ environments. In tissue engineering, the magnetic manipulation of cells is used in regenerative medicine^10^ for efficient cell loading inside scaffolds for bone tissues regeneration^11^ or to induce differentiation of MNP-laden embryonic stem cells^12^. Finally, MNP-mediated magnetic reprogramming of cellular function—magnetogenetics—is an emerging field in cell biology^13^ and neuroscience^14^.

MNP-based technologies enable the concentration and the manipulation of various bio-agents (BioA) both in-vivo and in-vitro. This is done by capturing the BioAs on selectively functionalized nanoparticle surfaces and then by guiding remotely the formed conjugates by external magnetic field. The superparamagnetic nanoparticles (SP-MNP) represent the most preferable choice for this kind of applications, due to their low aggregation probability and high magnetisation activated by external magnetic field.

Magnetic concentration, manipulation, and separation of BioAs is critically important for biosensing, and other in-vitro applications. The magnetic removal of BioAs from analysed solutions is highly selective, efficient, and often faster than centrifugation or filtration^15^. It enables efficient manipulations across microfluidics and enhances the sensors performance by removing the interfering species, increasing the concentration of BioA by orders of magnitude. The magnetic technologies are thus increasingly employed to concentrate and detect cells^16,17^ and molecular biomarkers^18,19^. Also, the realization of microfluidic devices based on the magnetic actuation of MNPs^20^ is expected to circumvent some critical issues in microfluidics, where the mixing and processing of fluids at the nanoscale become increasingly inefficient because of the dominance of capillary and viscous forces^21^.

The development of technologies for magnetic concentration and manipulation has progressed along different lines and approaches, depending on the specific problems which need to be addressed. It must be mentioned that the manipulation of superparamagnetic species on short scales, necessary for many biomedical applications, requires reasonably strong fields to align the SP-MNP magnetic moments, but also sharp magnetic gradients to provide strongly localized confinement of nanoparticles.

The application of electromagnets, both copper-based and superconducting, allows to achieve very high magnetic fields (> 1 T)^22^ but they are characterised by smooth spatial distribution, basically homogeneous on typical biological scales^23^. Permanent magnets, especially those having rectangular or needle-like shapes^19,24–26^, may generate relatively high fields (typically in sub-Tesla range) together with sharp magnetic gradients on mm and sub-mm scales^27,28^. Also, their cost is orders of magnitude cheaper than electromagnets and their handling and positioning is by far easier. Furthermore, advanced micro-scale patterning technologies allow to achieve enormous magnetic gradients in the close vicinity of the fabricated micromagnets^29–31^. Also, natural micro- and nanofeatures in continuous non patterned ferromagnetic films, such as magnetic domain walls (DW) can be used to manipulate magnetic particles^32^ and particle-protein conjugates^33^ on the micro-scales. Indeed, the choice of the magnet size, shape and strength depends on the specific application.

While the research on magnetic concentration and related manipulation of SP-MNPs has been strongly promoted in the biomedical field, the applications are not limited to it but have proved to be technologically efficient and cost-effective also in other areas, such as waste remediation, large-scale water purification, environmental monitoring, food production, industrial separation of minerals for mining and of metals for nuclear industry (see ref^15^ and reference therein).

In this paper we experimentally demonstrate the efficient confinement of small superparamagnetic nanoparticles with a Fe_3_O_4_ magnetic core by specifically tailored permanent magnets. The experimental data are in good agreement with employed modelling based on finite element analysis. We also define the conditions for a reliable confinement of nanoparticles as function of their diameters and for the motion of concentrated MNPs across microfluidic channels.

### Synthesis and characterization of MNPs

**Figure 1:**
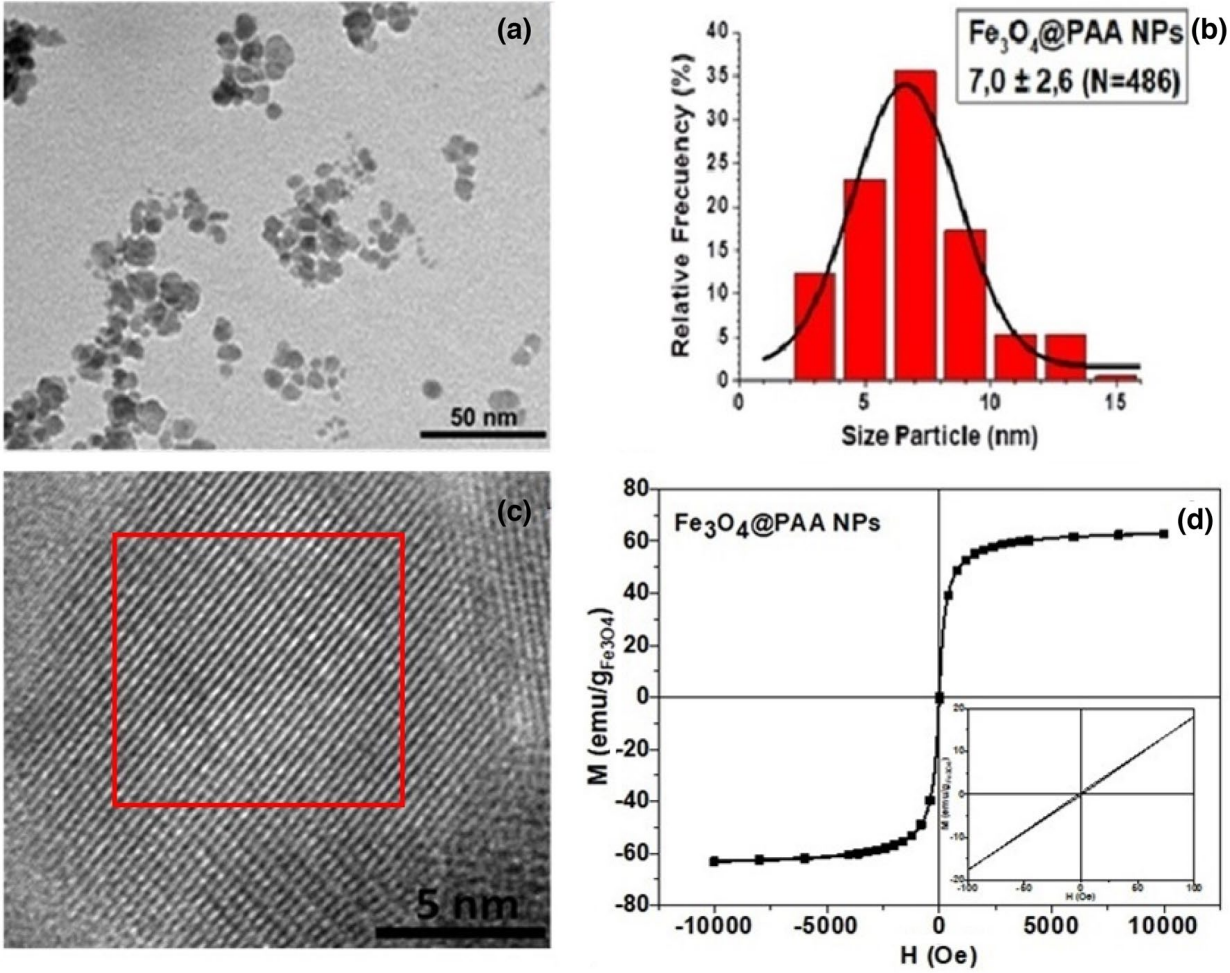
Fe3O4@PAA nanoparticle characterizations: (**a)** TEM image. (**b)** Statistical distribution of magnetic core diameters. (**c)** HRTEM image, where the red square denotes the area of highest crystalline quality. (**d)** Hysteresis loop at room temperature.

Single-core superparamagnetic iron oxide nanoparticles coated by polyacrylic acid (Fe_3_O_4_@PAA) were employed to investigate the magnetic confinement. The Fe_3_O_4_@PAA nanoparticles were obtained by co-precipitation, following Massart’s method^34^. The reaction was carried out for 1 h at 60 °C. The SP-MNP were characterized by different techniques to reveal their structural and magnetic quality. Transmission electron microscopy (TEM) micrographs were taken by JEOL JEM-1011 microscope operating at 100 kV and by LIBRA 200FE microscope for High-Resolution Transmission Electron Microscopy (HRTEM) operating at 200 kV. Direct current (DC) magnetization curves of the dried samples were measured using a vibrating sample magnetometer (VSM) (DMS, Lowell, MA, USA). TEM images (Fig. 1a) reveal an irregular spherical morphology with a medium magnetic core size distribution having a maximum at about 7 nm (Fig. 1b) and a total diameter of about 10 nm. HRTEM microscopy (Fig. 1c) discloses the crystalline nature of the iron oxide core with an inverse spinel structure, as proved by Selected-Area Electron Diffraction (SAED) analysis. A typical magnetization curve for the Fe_3_O_4_@PAA nanoparticles is shown in Fig. 1d. The curve has a standard superparamagnetic shape with vanishingly low magnetic hysteresis and negligible coercivity (H_C_ = 1.6 Oe). The saturation magnetization of 63 emu/g_Fe3O4_ is comparable with the highest values reported in literature for iron oxide SP-MNP^35^.

### Confinement tool

**Figure 2:**
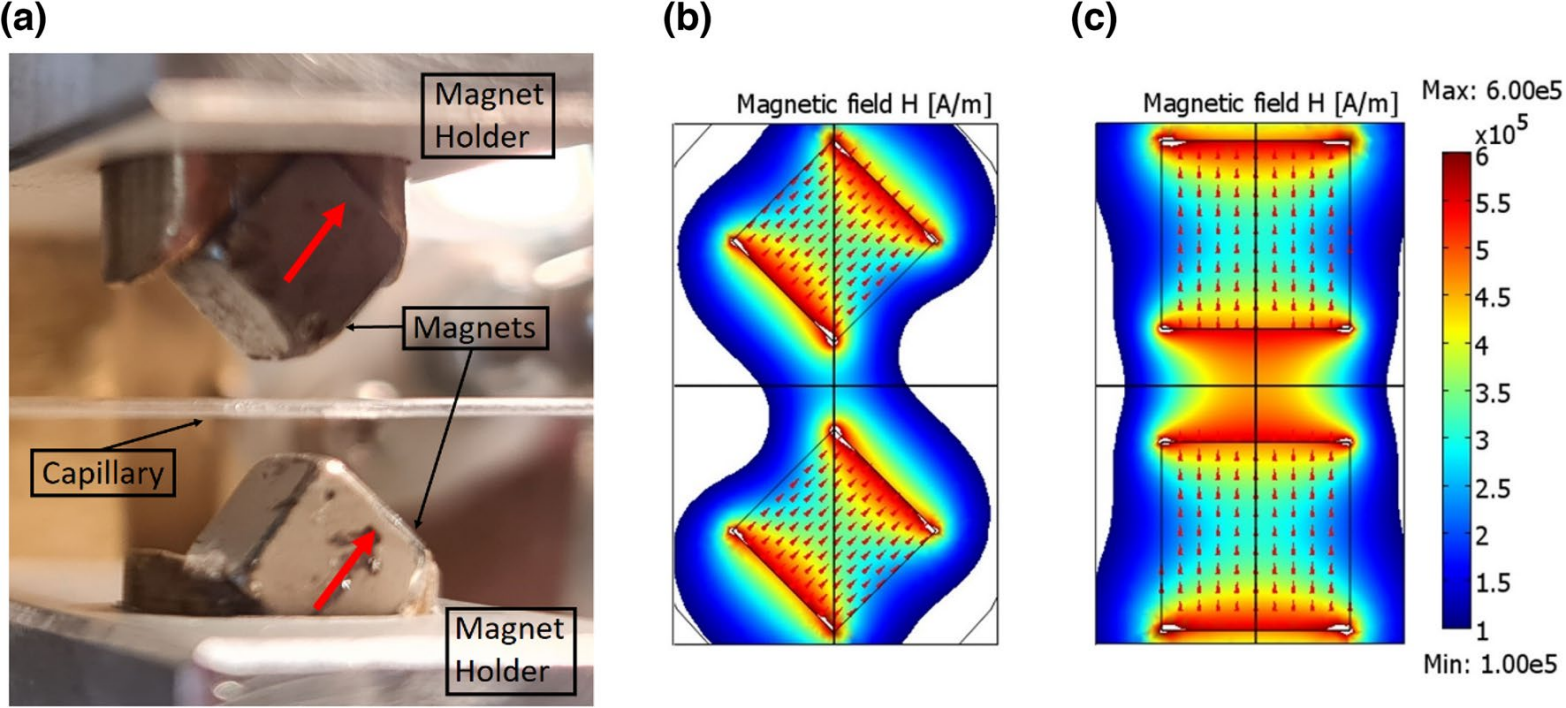
**(a)** Photo of the experimental MagD. (**b)** Calculated magnetic field distribution generated by the magnets in edge-to-edge configuration. For comparison in (**c)** we show magnetic field distribution simulated for magnets in face-to-face configuration. (**b,c)** Have a common colour scale for magnetic field values and the arrows show the directions of magnets magnetization.

A magnetic device (MagD) has been designed and fabricated to confine the SP-MNPs. The device consists of a quartz capillary placed between two NdFeB permanent magnets (Fig. 2a). The magnets are fixed by a fork-like holder and can be moved by a linear actuator along the capillary at a speed up to 120 µm/s. The capillary is loaded with MNP suspension by using a peristaltic pump connected by a silicon pipe. Direct observation of nanoparticle behaviour in quartz capillary under the effect of magnetic fields is performed by a microscope-connected CCD camera (Dino-Lite Pro, AnMo Electronics Corporation, Hsinchu City, Taiwan). Capillaries with diameters ranging from 500 to 3000 µm were used for the experiments—where the smallest size simulates microfluidic channels—and medical-grade silicone tubes (Silastic^®^ Rx 50 medical grade tubing) routinely used for intravenous therapies.

Considering the permanent magnets, our preference was given to rectangular shapes due to their expected sharp confinement geometry. To define their size and orientation, COMSOL simulations of cubic magnets with edges from 2 to 10 mm have been performed.

The 5 × 5 × 5 mm^3^ magnets were found sufficiently strong to bring the MNPs magnetisation close to saturation values. Next, we focused on the magnets orientation with respect to the capillary. The edge-to-edge geometry (Fig. 2a,b) was established as the optimal configuration, providing a significantly narrower distribution than the face-to-face one (Fig. 2c). The simulation of the magnetic field distribution for two 5 × 5 × 5 mm^3^ magnets distanced by 3 mm showed a clepsydra-like field distribution, with magnetic field reaching clear maxima (about 600 kA/m or about 7500 Oe) next to the magnets (red region). Yet, in the edge-to-edge case (Fig. 2b) the field is concentrated in a narrow sub-mm region with magnetic field of about 360 kA/m (about 4500 Oe) in the central point between the magnets, while in the face-to-face case (Fig. 2c) the generated field is broadly distributed with respective values of about 440 kA/m (about 5500 Oe). Even though both configurations generate magnetic fields able to bring the magnetisation of MNPs close to the saturation (see Fig. 1d), we have chosen the narrowest magnetic distribution to concentrate the nanoparticles in the smallest possible volume. The vertex-to-vertex configuration was also simulated and ruled out, because in spite of quite narrow field distribution it was characterized by lower magnetic field intensity.

The complete set of calculated parameters and a detailed explanation is presented below in the discussion part.

## Experimental section: confinement and manipulation of MNP

**Figure 3:**
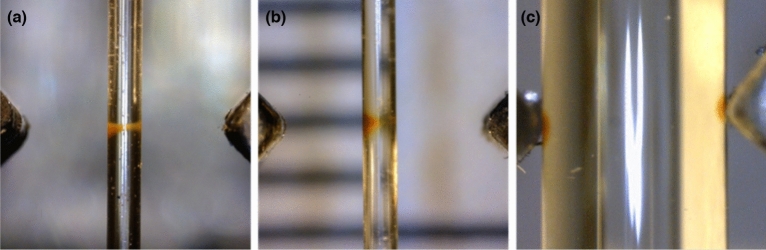
Experimental realization of MNP confinement by permanent magnets separated by 3 mm. (**a)** A 500 µm wide capillary is placed at the mid-point between magnets: MNPs are concentrated between the magnets in a clepsydra-shaped region spanning across the entire capillary section. (**b)** The 500 µm wide capillary is displaced by 100 µm from the midpoint to the left: MNPs coalesce into a blob-like configuration next to the nearest magnet. (**c)** For a 3 mm wide capillary (occupying the entire gap between magnets), the MNPs coalesce into dense blobs next to the magnets.

To investigate the confinement of the employed nanoparticles in our magnetic tool, we injected a 50 µg/mL concentrated suspension of MNP in the 500 µm wide capillary placed precisely at the midpoint between magnets. The MNP suspension represents a small part of the circulating liquid consisting mainly of the de-ionized water (18 MΩ‧cm) and is kept separated from it by two air gaps (at about 5 cm from each other) to avoid mixing and subsequent dilution. As can be seen in Fig. 3a, the nanoparticles get concentrated (in ca.10 min) into a stable configuration resembling the magnetic field distribution simulated by COMSOL (Fig. 2b). It consists of two lobes at the capillary wall next to the magnets connected by a narrow “bridge”: essentially, a clepsydra-shaped configuration (CSC) (Fig. 3a). This shape results from a joint effect of the magnetic field and the spatial constraints produced by the capillary.

This configuration appears very sensitive to any displacement of the capillary from the central symmetrical position between the magnets: shifting the capillary to either left or right, a blob-like droplet of MNPs forms at the capillary wall next to the closest magnet. This transition depends on the capillary size and on the distance between the magnets. For example, for a 500 µm wide capillary and magnet separation of 3 mm even a 100 μm displacement of the capillary to the left (Fig. 3b) is sufficient to break the CSC, while for magnet distancing of about 8 mm the clepsydra is stable even for displacements up to 200 µm. Indeed, moving the capillary along the line connecting the magnets, the MNPs can sense regions where the magnetic energy is significantly higher than at the midpoint and are attracted there. Accordingly, maintaining the same concentration of nanoparticle and the 3 mm gap between the magnets, the employment of a capillary as wide as the separation distance leads the MNPs to concentrate in two dense blobs near the edges of magnets (Fig. 3c).

**Figure 4:**
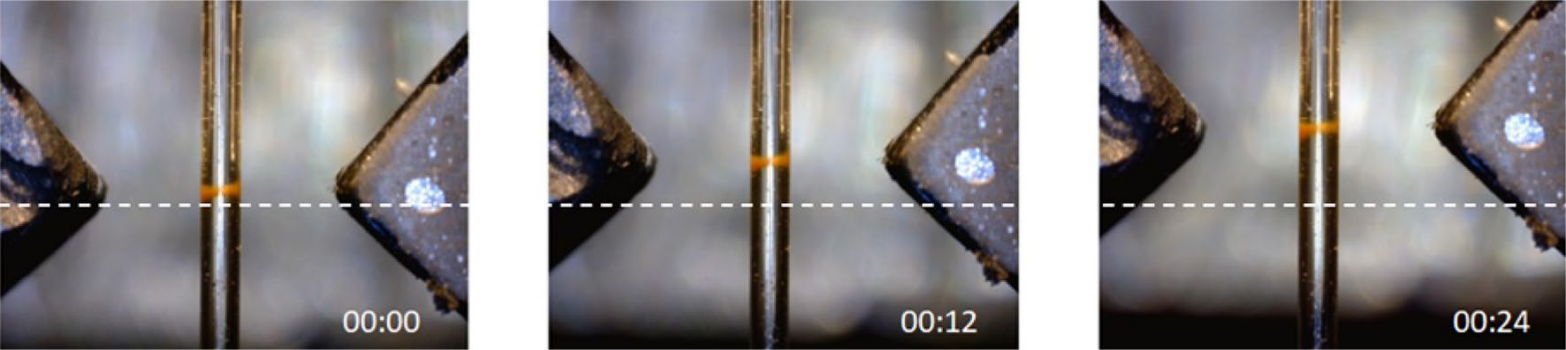
Dynamic behaviour of CSC. It solidly follows the magnets movement along the capillary while keeping its shape. Magnets speed: 60 µm/s; gap 3 mm; capillary 500 µm wide. For sake of clarity, a dashed line has been superimposed as a reference. Time is given in mm:ss format and 00:00 corresponds to initial magnets position after CSC formation.

The CSC can remain stable even when magnets are moved along the capillary. It solidly follows the magnets along the capillary for a range of magnets speeds and separations. Figure 4 shows the example of such motion for the prototypical configuration, 500 µm capillary and 3 mm magnet separation, at 60 µm/s speed. We have further investigated the effect for magnets speed 30, 60, 120 μm/sec and separation between magnets of 3, 5, 6, 8, and 10 mm to define the “stability window” for the motion of confined MNPs.

**Figure 5:**
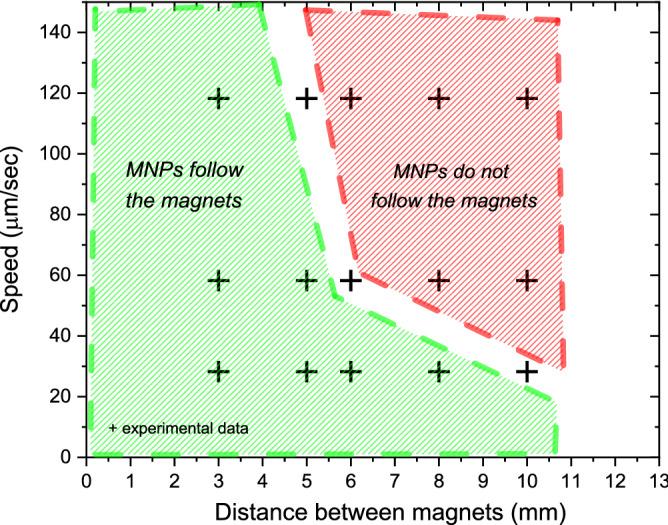
Stability window for the CSC upon translation in the 500 µm capillary. Crosses mark the experimental data while green and red regions delimitate the parameter spaces where a CSC solidly follows the magnets or not, respectively. The data points corresponding to 5 mm to 120 µm/s, 6 mm to 60 µm/s, 10 mm to 30 µm/s gave not reproducible experimental results in terms of stability and were excluded from the green/red regions.

This stability window is represented by the green area in Fig. 5. Clearly, a strong correlation exists between the magnets separation and the highest speed at which the confined MNPs can solidly follow the magnets: the higher magnets separation the lower speed necessary to break the confinement. For magnets distanced by 10 mm the MNPs can be rigidly moved only at 20 μm/s, while for separation smaller than 5 mm, the MNPs shape can be maintained even at the highest available speed: 120 μm/s. All the experiments were done using the MNP concentration of 50 µg/mL. At one selected data point, namely at 3 mm and 30 µm/sec, lower concentrations of 10 µg/mL and 3 µg/mL have been also investigated, revealing no dependence of the CSC stability on the concentration.

## Modelling and discussion

To decrease the size of the magnetic particles from micro- to nanometres would be highly beneficial and would enable innovative high-throughput purification/separation methodologies. Yet, the broad use of nano-sized particles has been so far hindered because it is increasingly difficult to control by magnetic fields small sub-100 nm nanoparticles against the randomizing effects of thermal fluctuations. This has put significant limitations to magnetic manipulation. Large nanoparticles typically have magnetic remanence and thus a strong tendencies towards aggregation, whilst the control would be markedly facilitated in the superparamagnetic regime, induced by a size-dependent transition below a material-dependent critical diameter (about 20–25 nm for Fe_3_O_4_) and characterized by no remanent magnetization at zero field^36–38^. Also, from a biotechnological point of view, the use of MNPs with sizes comparable to biological entities (for example, proteins (5–50 nm) or viruses (> 20 nm)) is highly advantageous.

To analyse and describe the experimental confinement data we must compare the magnetic and thermal energies acquired by SP-MNPs in our experimental conditions. Assuming the situation of thermal equilibrium, a magnetic particle is captured by the magnetic field in a region where the energy of randomizing thermal fluctuations is lower than the magnetic energy. The thermal energy of the particle at room temperature (T = 300 K) is given by

1 $${E}_{T}=\frac{3}{2}{k}_{B}\cdot T\approx 6\cdot {10}^{-21}\mathrm{ J}$$

where *k*_*B*_ is the Boltzmann constant and *T* is temperature.

The magnetic energy can be expressed as the scalar product of the particle magnetic moment ${{\varvec{m}}}_{p}$ and the applied magnetic field ***H***^39^:

2 $${E}_{m}=\frac{1}{2} {\mu }_{0}{{\varvec{m}}}_{p}\cdot {\varvec{H}}=\frac{1}{2}{\mu }_{0}({{m}_{p}}_{x}{H}_{x}+{{m}_{p}}_{y}{H}_{y}+{{m}_{p}}_{z}{H}_{z}),$$

where ${\mu }_{0}$ is the magnetic permeability of vacuum. Here the magnetisation of the water is considered negligible. Knowing the magnetic field generated by permanent magnets, we can define the magnetic moment of the MNPs at all the coordinates by using the experimental magnetization curve (Fig. 1d) and converting the magnetic units by:

3 $${{\varvec{m}}}_{p}\left[A{m}^{2}\right]={{\varvec{M}}}_{m}\left[\frac{emu}{g}\right]{\rho }_{m}\left[\frac{g}{{cm}^{3}}\right]{V}_{m}\left[{cm}^{3}\right]\cdot {10}^{-3}$$

where *M*_*m*_*, ρ*_*m*_*, V*_*m*_ are magnetization (from magnetization curve), density and volume of the particle magnetic core, respectively. The detailed map of the spatial distribution of the magnetic energy of MNPs in the experimental configuration of the MagD is obtained from FEM simulations. The magnetic energy, *E*_*m*_, is calculated applying Eq. (2) and is compared with the thermal energy from Eq. (1), defining the conditions and areas where E_m_(H) > 6 × 10^−21^ J. The simulations are performed for spherical nanoparticles with 8 nm diameter of the magnetite core. This value is taken assuming that the magnetic confinement and dynamics is dominated by the 7 and 9 nm large MNPs (see Fig. 1b and discussion below).

**Figure 6:**
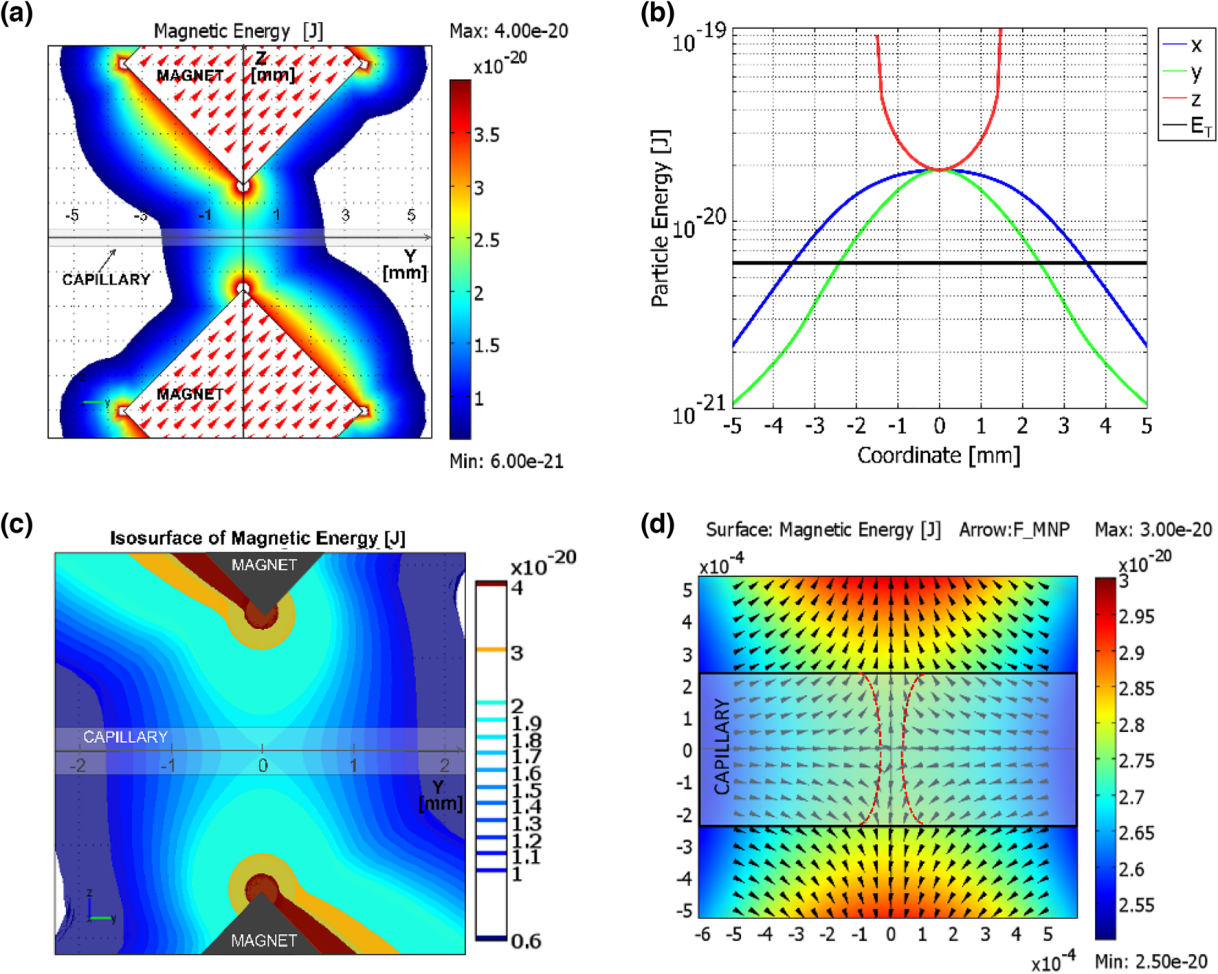
**(a)** The distribution of the magnetic energy in yz-plane for the experimental set-up (Fig. 2a,b). The color scale depicts the 0.6–4.0 × 10^–20^ J energy interval where E_m_ > E_T_. The red arrows show the directions of magnetization in the magnets. (**b)** Magnetic energy distribution along the axes defined in **(a)** (x-axis orthogonal to the plane of the figure), while the black line represents the thermal energy. (**c**) Zooming of (**a**) with the isosurfaces of magnetic energy between the magnets. (**d**) The further zooming of the working area to compare the magnetic energy maps with the confinement of nanoparticles from Fig. 3a, sketched by two red dashed lines. The black arrows show the direction of the magnetic forces acting on nanoparticles.

The results of these numerical simulations are shown in Fig. 6. It indicates the yz-plane distribution of the magnetic energy of nanoparticles via a colour map, where the colour scale starts from the thermal energy level. Namely, the white background corresponds to regions where the thermal energy dominates; the coloured regions to the area where magnetic confinement occurs. Figure 6b shows the magnetic energy distribution along the three axes of coordinates in comparison with the thermal energy indicated by a black horizontal line. The MNP magnetic energy exceeds the thermal energy for all the points along z-axis, for y <  ± 2.4 mm and for x <  ± 3.5 mm.

The magnetic energy, as shown in Fig. 6a, has a clepsydra-like profile: along the y-axis, the energy has a clear maximum centred in the gap between the magnets while along the z-axis presents two maxima at the magnet edges and a broad minimum in the middle. In the region around the magnets where the magnetic energy is higher than the thermal one, the randomizing effect of the Brownian motion is overcome by the stabilizing effect of the magnetic field. Once magnetic nanoparticles enter in that region they cannot be displaced by thermal fluctuation and remain confined there.

The clepsydra shape is additionally emphasized in Fig. 6c. The colour maps in Fig. 6c are split in magnetic energy isosurfaces, useful for a better understanding of the MNP magnetic dynamics. For a better comparison of the calculations and experimental data, in Fig. 6d we zoom further on the area occupied by the capillary, where the confining energy maps are compared with the distribution of nanoparticles from Fig. 3a, sketched by two fitting red curves and the black arrows indicate the direction of the magnetic forces locally acting on MNPs. The experimental clepsydra is confined within approximately a 200 µm area at the capillary walls, and restricted to below 100 µm in the centre. This looks somehow narrower than the clepsydra-like isosurface with the highest energy (1.9 × 10^–20^ J) which still fits inside the capillary.

The explanation of this apparent deviation can be given both qualitatively and quantitatively. The qualitative explanation is backed by the distribution of the magnetic forces shown in Fig. 6d. Inside this isosurface the magnetic forces push the nanoparticles towards the central vertical axis, while the round-like shape is provided by the rotation of the gradual magnetic forces when approaching the capillary wall. Quantitatively, the explanation of the shape of the CSC is in agreement with thermodynamical models on confinement of Brownian particles by external forces^40,41^. It has been shown that the strict confinement is realised in areas where the deep potential (magnetic energy in our case) is about 4 times higher than the thermal energy. This is in good agreement with experimental and calculated data presented in Fig. 3 and Fig. 6d, where the confinement is realised for E_m_ (H) ≥ 1.9 × 10^–20^ J, which is about 3.2 times over the thermal energy.

It is worth highlighting here, that the MNP solution is physically constrained by the capillary, which is where the MNPs can be confined in a stable configuration not coalescing into the energy maxima located at the edges of magnets (Fig. 3c).

We now expand our calculations for MNPs from same magnetic material but different diameters of the magnetic core. The energy *E*_*m*_ of nanoparticle is directly proportional to its magnetic moment m_p_ and the applied field *H,* where *m*_*p*_ is proportional to the particle magnetization, its density and its volume (Eqs. (2) and (3)). Thus, the ratio of magnetic energy for two MNPs having magnetic diameters d_p1_ and d_p2_ is:

4 $$\frac{{E}_{m2}}{{E}_{m1}}= \frac{{d}_{p2}^{3}}{{d}_{p1}^{3}}$$

**Figure 7:**
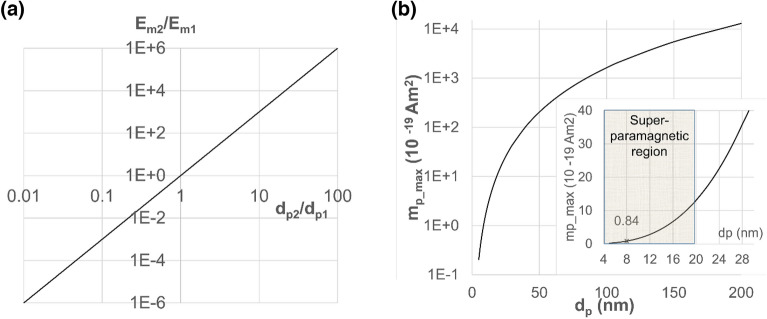
(**a**) Ratio of magnetic energy E_m_ for two similar MNPs having magnetic diameters d_p1_ and d_p2_ in the same magnetic field. (**b**) Maximal magnetic moment of the particle m_p__max versus its magnetic diameter d_p_.

This gives a linear dependence in logarithmic coordinates shown in Fig. 7a. It physically means that, using MNPs with larger magnetic diameter, the *E*_*m*_ scales as the third power leading to an enlargement of the area where the MNP are magnetically trapped. Yet, as superparamagnetic-ferromagnetic transition happens at around 20 nm, inter-particle aggregation effects must be considered for diameters exceeding that value.

Figure 7b shows the maximum magnetic moment *m*_*p_max*_ achievable in the superparamagnetic regime for employed iron oxide nanoparticles. Assuming a magnetic field sufficient to saturate the particle magnetization 60 emu/g and a density of 5.2 g/cm^3^, the magnetic moment is m_p_max_(Am^2^)≈1.6 × 10^–22^·d_p_(nm)^3^ (Fig. 7b). For example, for 8 nm MNPs the magnetic moment can achieve m_p_max_(Am^2^) = 0.84 × 10^–19^ Am^2^. For the largest superparamagnetic 20 nm nanoparticle we can achieve the maximum magnetic moment of about 13 × 10^–19^ Am^2^.

Summarizing, we showed that, via a proper selection of permanent magnets (considering their shape, orientation, distancing and magnetic intensity), it is possible to achieve high and sharply confined magnetic fields, able to trap and control small SP-MNP on sub-mm scales. We accurately define the conditions at which the magnetic energy exceeds the destabilizing thermal kinetic energy of the nanoparticles, as function of external magnetic field, particle magnetisation and particle diameter.

While our experimental data cannot rule out the formation of aggregates or chains of MNPs in the MagD device^42,43^, our theoretical analysis demonstrates that their existence is not essential for magnetic confinement and control. Indeed, our experimental data are fully explained by a single-particle model where the MNPs act independently in the magnetic field. Moreover, straightforward calculations demonstrate that the magnetic fields generated by the presented magnetic configuration (Figs. 2 and 6) can trap superparamagnetic nanoparticles as small as 7–8 nm, in contrast with the previously proposed 50 nm lower bound for the control of magnetic nanoparticles by external magnetic fields^43^. Our results also indicate that the confinement of extremely low concentrations of nanoparticles, orders of magnitude below the visualization level, is possible. It would require a reasonably high magnetization of the MNPs, like the one shown in Fig. 1d, which is nevertheless reasonable for state-of-art magnetite iron oxide nanoparticles. Finally, there is an excellent agreement between the experimentally detected and the calculated clepsydra-like shapes, the latter resulting from a joint magnetic and physical (by the capillary) confinement of the nanoparticles.

From Eq. (2) we can also say that for whatever small diameter of a magnetic nanoparticle, there exist a magnetic field H able to induce a magnetic energy exceeding the thermal energy. Our experiments show that standard commercial permanent magnets are well suited to control sub-10 nm nanoparticle, while much smaller nanoparticles (based on the same material) may require fields hardly achievable in laboratory conditions.

Considering the CSC stability during motion (Fig. 5), a comprehensive explanation would require an accurate knowledge of the hydrodynamic diameter of the MNPs. This parameter is typically measured by Dynamic light scattering (DLS), which however leads to considerable overestimations of the diameter of the nanoparticles due to the effects of multiple scattering and the assumption of spherical particles^44^. Indeed, the DLS measurements performed on these nanoparticles, characterized by a total diameter of 10 and a magnetic core with a diameter of 7–8 nm (see above), gave hydrodynamic diameters of 65 ± 29 nm. Both the size overestimation and the substantial error do not allow, in our opinion, to correctly interpret the experimental data in Fig. 5. A more accurate definition of the hydrodynamic diameter is possible from direct measurements of the diffusivity of MNPs currently under investigation. We anticipate nevertheless that the stability of the CSC during motion should be more instable at low MNPs concentrations, in pure single particle regimes, while possible aggregates with large magnetic moments would solidly follow the motion of magnets.

Finally, we would like to underline that the possibility to individually control ultra-small nanoparticles is crucial for bio-applications, enabling the one-to-one capture of various proteins or viruses, typically in the same range of sizes. Such capability is critical in diagnostics, where the magnetic labelling can allow not only for the detection of selected bio-agents by magnetic sensors but can also permit their statistically accurate quantification.

## Conclusions

An experimental set-up—consisting of properly shaped permanent magnets and a capillary system—was employed to confine small superparamagnetic nanoparticles in sub-mm spatial shapes. We also demonstrate that it is possible to move the whole batch of nanoparticles with speeds depending on the distance between magnets. We give a mathematically accurate description of the confinement of nanoparticles by magnetic field, and define the conditions for which the magnetic energy overcomes the Brownian motion. Our experiments and calculations indicate that magnetic systems based on standard commercial permanent magnets can be successfully used for the trapping and manipulation of SP-MNP for various applications.

## Data Availability

Correspondence and requests for material should be addressed to AS, TS and VAD.
